# Ocular Infection Preceding Major Epidural Abscess

**DOI:** 10.1155/2014/245013

**Published:** 2014-09-30

**Authors:** Liam Dunbar, Ryan Johnstone

**Affiliations:** Hutt Valley DHB, Private Bag 31907, Wellington, Lower Hutt 5040, New Zealand

## Abstract

Staphylococcal bacteremia is an important clinical entity. A 74-year-old lady presented with an isolated staphylococcal ocular infection; this was treated with a short course of antibiotics, a prolonged course of steroids, and discharge from hospital with outpatient clinic followup. She represented three weeks later to the emergency department with back pain, raised inflammatory markers, and positive blood cultures. On magnetic resonance imaging (MRI), an extensive epidural collection was seen. This was surgically decompressed, and she was treated with appropriate intravenous antibiotics. Despite a complicated postoperative course, she made an excellent recovery. This case reviews the important clinical and radiological features of the presentation of a major epidural abscess and it also suggests a potentially unusual primary source. The clinician is reminded to always have a high index of suspicion regarding staphylococcal bacteremia and the potential for seeding to the epidural space.

## 1. Introduction

Staphylococcal bacteremia is an important clinical entity, with high mortality if left undetected and untreated. Complications of staphylococcal bacteremia are often difficult to identify initially and this leads to a delay in treatment with often detrimental clinical sequelae. Complications are known to include endocarditis, vertebral osteomyelitis, and epidural abscess [1]. With regard to hematogenous spread, infection of the skin, soft tissues, respiratory tract, and the urinary tract are the usual primary sources [1]. When the diagnosis is made promptly and surgical intervention carried out without delay, permanent neurological deficit can be avoided. We present a case of major epidural abscess in the context of staphylococcal bacteremia, reviewing the important clinical and radiological findings and highlighting lessons to the clinician.

## 2. Case Presentation

A 74-year-old lady with no medical comorbidities presented with reduced visual acuity. Vitreous cultures prior to antibiotic administration grew multisensitive* Staphylococcus aureus*. Three sets of blood cultures were taken prior to commencement of antibiotics and were negative. She received treatment with intravenous flucloxacillin initially; however, a repeat vitreous sample was clear and intravenous antibiotics were stopped. A course of oral antibiotics was completed. She was continued on a course of oral prednisone for endophthalmitis with continued ophthalmology outpatient followup and discharged from hospital after a weeklong admission. She presented three weeks later with a four-day history of insidious onset lumbar back pain. This was accompanied by rigors, fevers, and anorexia.

Initial examination revealed boggy swelling over the lumbar spine with midline and paraspinal tenderness to palpation. She had symmetrical range of motion of the spine and a normal neurological exam.

X-rays of the lumbar spine revealed an old L3 compression fracture and an L4/L5 spondylolisthesis. Initial blood tests showed a white cell count of 13, neutrophils of 12.7, and C reactive protein (CRP) of 329.

The triad of back pain, raised inflammatory markers, and possible recent* Staphylococcus* infection led to potential concern of an epidural abscess. MRI with gadolinium was requested.

**Figure 1:**
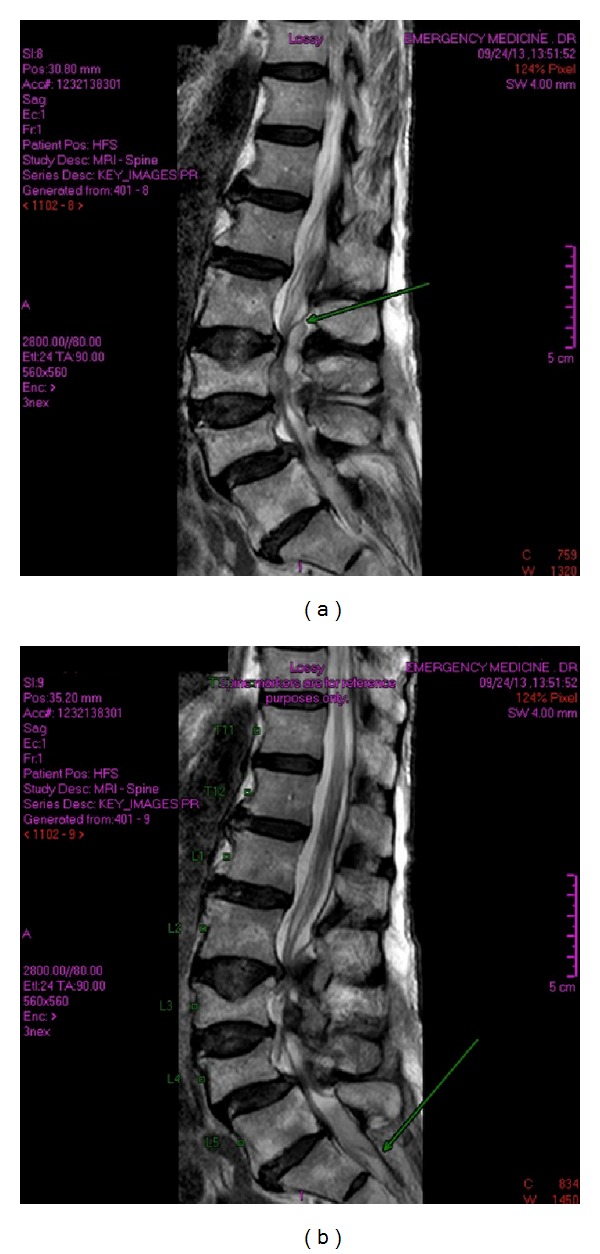
(a) T2 weighted image. A 2-3 mm anterolisthesis of L4 with respect to L5 is demonstrated; vertebral bodies are otherwise normally aligned. Increased signal intensity and a wall enhancing fluid collection are seen in the posterior epidural space. This extends from L2 to S1; the arrow depicts the upper extent. (b) T2 weighted image. Loss of vertebral body height and increased endplate concavity at T9, L3, and L4. Accompanying linear areas of signal loss are characteristic of fractures parallel to the superior end plates of T9 and L4. An anterior epidural abscess component is shown from L3 to L4. Posterior extension of the abscess is shown to S1 level as indicated by the arrow.

**Figure 2:**
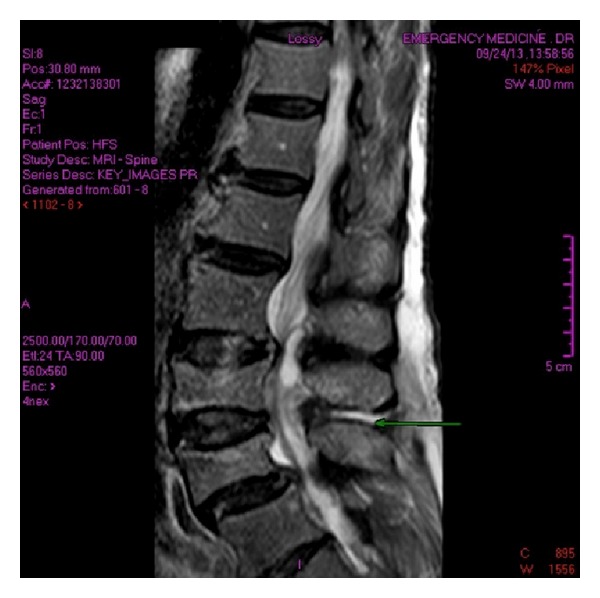
STIR (short-inversion-time inversion recovery) sagittal image. Increased fluid signal intensity is noted in the interspinous region of L3/L4 consistent with extension of infection.

**Figure 3:**
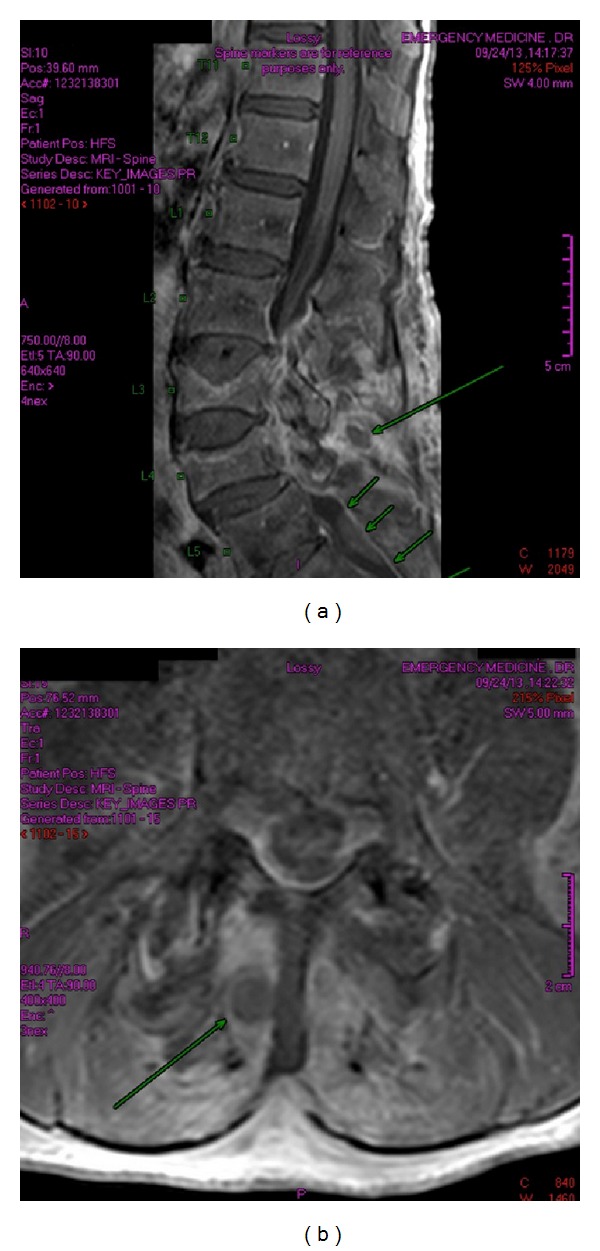
T1 weighted images postgadolinium sagittal and axial views: enhancement and oedema shown in the posterior ligamentous complex.

The paraspinous region demonstrates at least two small collections (to the right of the L4 spinous process as depicted by the large arrow on both images of Figures 1(a) and 1(b)).  Figure 2 demonstrates the extension of the infection. Significant compression of the thecal sac and spinal cord is seen on Figure 3(b), along with the collection opposite the L4 spinous process.

Subsequent blood cultures grew multisensitive* Staphylococcus aureus*. She was taken to the operating theatre for posterior lumbar decompression of L2 to S1 with open washout and evacuation of an epidural abscess. Nonoperative management was not considered. The indication for surgery was significant sepsis coupled with the radiological extent of the abscess with anterior and posterior elements exerting significant mass effect upon the thecal sac.

She was commenced on intravenous flucloxacillin for a total of six weeks. Abscess cultures grew multisensitive* Staphylococcus aureus* as did prior vitreous cultures. A transesophageal echocardiogram ruled out endocarditis. Her postoperative course was complicated by syndrome of inappropriate antidiuretic hormone secretion (SIADH) and hyponatremia; postoperative anemia; and clostridium difficile diarrhea secondary to antibiotic administration. She was discharged home after a two-week admission. Upon discharge, weekly reviews were carried out in outpatients with repeated inflammatory markers. She had an excellent response to the therapy; her pain settled completely and inflammatory markers returned to normal limits within four weeks of intravenous treatment. A total of six-week intravenous antibiotics were completed.

## 3. Discussion

The diagnosis of epidural abscess represents a challenge; changing neurology is preceded far in advance by nonspecific symptoms of back pain, fever, and localized tenderness [1]. Importantly, early diagnosis is the key prognostic factor [1]. The classic triad of back pain, fever, and neurological deficit is present initially in only 10–15% of patients [1, 2]. As abscess, formation grows symptoms progress to radicular pain, reflex changes, bladder and bowel change, and ultimately paralysis which is quickly irreversible. Thus, the need for rapid diagnosis and treatment cannot be over emphasized [3].

In 50% of cases infection reaches the epidural space through hematogenous seeding, and in 30–40% the source is never identified [4, 5]. Skin, soft tissues, respiratory, and urinary tract are the usual sources [1]. Interestingly, the current literature does not comment on ocular infection as a primary site of bacterial entry. However, endophthalmitis is a known complication of epidural abscess, with a poor prognosis for visual outcomes [6].

MRI with gadolinium has established itself as the imaging modality of choice. It has greater than 90% specificity and sensitivity for detecting spinal epidural abscess [7–9]. Abscess material and the spinal cord have the same intensity on T1 weighted images [1]. In T2 weighted images, the area of abscess usually shows increased signal. Fat saturated images give information on bone marrow/soft tissue involvement and subsequent extent of the infection [1].

Surgical treatments' aims are twofold to decompress the spinal column to prevent neurological deterioration and to eradicate the source of sepsis [1]. Accompanying treatment with* Staphylococcus* sensitive intravenous antibiotics is mandatory with a usual course of four to six weeks. The literature states the mortality for epidural abscess to be 2–20%, but untreated* Staphylococcus *bacteremia may be as high as 80% [1, 4, 5, 10]. Factors relating to poorer outcome include patients with multiple comorbidities, immunosuppression, and growth of multidrug-resistant organisms [4, 5].

## 4. Conclusion

This case suggests the possibility of an unusual primary source of infection leading to epidural abscess. Interestingly, our patient presented remarkably well despite an extensive epidural collection. Prompt surgical drainage and decompression coupled with appropriate antibiotic therapy led to an excellent outcome. We are unable to ascertain if the untreated ocular infection led to* Staphylococcus *bacteremia or if the* Staphylococcus *bacteremia was present from the outset from another source. The key principles remain early diagnosis and expeditious surgical treatment. A high degree of clinical suspicion is required in the absence of focal neurological findings.* Staphylococcus *bacteremia or even localized staphylococcal infection should alert the physician to keep the possibility of epidural seeding in mind.
